# A Narrative Review of the Role of Non-Viral Circulating Tumor DNA Profiling in Predicting the Treatment Response and Recurrence in Head and Neck Squamous Cell Carcinoma

**DOI:** 10.3390/cancers17142279

**Published:** 2025-07-09

**Authors:** Ugur Gezer, Rasim Meral, Emre Özgür, Ebru. E. Yörüker, Abel Bronkhorst, Stefan Holdenrieder

**Affiliations:** 1 Department of Basic Oncology, Oncology Institute, Istanbul University, 34093 Istanbul, Türkiye; ugurd@istanbul.edu.tr (U.G.); emre.ozgur.86@istanbul.edu.tr (E.Ö.); akisik@istanbul.edu.tr (E.E.Y.); 2 Department of Radiation Oncology, Oncology Institute, Istanbul University, 34093 Istanbul, Türkiye; rasim.meral@istanbul.edu.tr; 3 Institute of Laboratory Medicine, TUM University Hospital Munich, German Heart Center, 80636 Munich, Germany; bronkhorst@dhm.mhn.de

**Keywords:** head and neck squamous cell carcinoma, ctDNA, treatment, radiotherapy, chemoradiation, immunotherapy

## Abstract

Head and neck squamous cell carcinomas (HNSCCs) that develop from the mucosal epithelium in the oral cavity, pharynx, and larynx are a heterogeneous group of malignant tumors. A lack of appropriate screening and diagnostic methods leads to late diagnoses, with the majority of patients having locally advanced disease, which is associated with a high risk of local recurrence and a poor prognosis and is usually treated with combination therapies. Biomarkers for predicting the therapy response and risk of recurrence in HNSCC patients are urgently needed. Liquid biopsy, e.g., the profiling of circulating biomarkers in bodily fluids, is a promising approach with increasing utility in the early detection and diagnosis of cancer, monitoring cancer progression, patient stratification and treatment selection, detecting minimal residual disease (MRD), and predicting recurrence across different cancer types, including HNSCC. Among liquid biomarkers, circulating tumor DNA (ctDNA), which is based on detecting tumor-specific mutations, insertions/deletions, copy number alterations, and methylation, is the most promising transformative tool in cancer management and personalized cancer treatment. In this review, we provide an update of recent data on the role of non-viral ctDNA in the management of HNSCC patients. Accumulating data suggests the enormous potential of ctDNA profiling by serial sampling during and after definitive therapy in detecting MRD and predicting recurrence in HNSSC patients treated with a single treatment modality (surgery or radiotherapy) or with combination therapies, including immune-checkpoint-inhibitor-based immunotherapy. By incorporating the latest immunotherapy trials and organizing the data by the treatment modality, this review offers a novel perspective not found in previous surveys.

## Simple Summary

Circulating tumor DNA (ctDNA)-based liquid biopsy is increasingly being integrated into the clinical management of patients with various cancers. Several studies have demonstrated the clinical utility of longitudinal ctDNA analysis in the management of patients with head and neck squamous cell carcinoma (HNSCC). Applications include the detection of minimal residual disease (MRD) to predict recurrence in HNSCC patients after curative surgery, and the assessment of early response and recurrence to radiotherapy (RT) or combination therapies such as chemoradiotherapy ± surgery in patients with locally advanced HNSCC. Recent studies indicate the promising potential of ctDNA in assessing the efficacy of immune checkpoint inhibitor-based immunotherapy in advanced HNSCC. Well-designed, prospective validation studies could provide high-level evidence for the use of ctDNA in routine care, with the aim of reducing mortality and morbidity in HNSCC patients in the future.

## 1. Introduction

Head and neck cancer (HNC) is an umbrella term for a group of heterogeneous malignant tumors that arise in the salivary glands, nasal cavity, paranasal sinuses, oral cavity, nasopharynx, oropharynx, hypopharynx, and larynx. Although various histological tumor types are found in the head and neck region, more than 90% of HNCs are squamous cell carcinomas (HNSCCs) that develop from cells lining the mucosal epithelium of the oral cavity, pharynx, and larynx [1]. HNC is one of the most prevalent cancers, accounting for about 7% of all cancers and almost 5% of all cancer-related deaths [2]. Over the next two decades, human papillomavirus (HPV)-related oropharyngeal cancer is expected to account for the majority of all HNC cases. Conventional imaging techniques, including high-resolution ultrasound, computed tomography, and magnetic resonance imaging, are used to detect HNSCC. However, these image-guided techniques provide only indirect evidence of the suspected lesion and eventually require confirmation by a tissue biopsy [3,4].

Only a subgroup of HNSCC patients (approx. 30–40%) have early-stage disease at diagnosis, while the majority of patients (above 60%) present with locally advanced disease [5,6]. In patients with early-stage disease, surgery or radiotherapy (RT) alone is the main treatment option [7]. Locally advanced HNSCC, defined as T3– or N1–3, is associated with a high risk of local recurrence and a poor prognosis [8]. Treatment options for these patients include the combination of RT, chemotherapy (CTx), targeted therapy, and immunotherapy, even if the choice of optimal treatment remains controversial [9]. A re-evaluation of 126 randomized, controlled clinical trials showed that combination treatments consisting of concurrent RT with nimotuzumab or conventional concurrent chemoradiotherapy (CRT) had better efficacy and a greater survival benefit than RT alone, without increasing the side effects [9].

The lack of appropriate screening and diagnostic methods is one of the main reasons for the delay in the early diagnosis of HNSCC. In addition to late diagnoses, there is a lack of validated biomarkers for prognoses and the prediction of treatment responses based on specific, non-invasive, and reliable methods for patients with HNSCC. Thus, disease recurrence, metastases, and drug resistance lead to poor outcomes in these patients, with unsatisfactory 5-year survival rates [10]. It is increasingly evident that genetic and genomic analyses are useful tools to stratify patients for treatment, monitor the tumor response to cancer therapy, and detect disease progression as soon as possible. In this sense, liquid biopsy is an expanding field of research that is increasingly being integrated into the management of cancer patients.

## 2. Liquid Biopsy

Circulating cell-free nucleic acids refer to DNA and RNA fragments of various sizes that occur in bodily fluids, such as serum or plasma, saliva, urine, and pleural, peritoneal, and cerebrospinal fluids [11]. They can exist free/unbound, enclosed in a vesicle, or as complexes of DNA bound to proteins as nucleosomes [11]. Nucleic acids are released from cells into circulation via several mechanisms, with apoptotic and necrotic cell death as the main mechanisms [12]. Various aspects of circulating DNA and RNA have been the subject of research interest in the last two decades: including the analysis of total cell-free DNA (cfDNA), tumor-derived cfDNA, methylated DNA, cfDNA fragmentation patterns (i.e., fragmentomics), circulating nucleosomes, various classes of RNA molecules, and extracellular vesicles such as exosomes. Besides circulating tumor DNA (ctDNA) analysis, methylation-based-tumor DNA analysis, fragmentomics, and exosomal markers are promising markers of liquid profiling. ctDNA refers to the fraction of cfDNA derived from tumors released into bodily fluids either through cell death mechanisms, such as apoptosis or necrosis, or via active secretion by tumor or surrounding cells. [13]. In most cases, ctDNA constitutes only a small part (<1%) of the total plasma DNA, whose largest proportion derives from hematopoietic cells and other tissues of the body. As the impairment of phagocytosis in the tumor microenvironment leads to the increased release of genetic material from dying cells, the anatomic location of the tumor could affect ctDNA levels in different fluids [14], which is also affected by different treatment modalities, such as surgery, RT, and CTx. Ongoing research on ctDNA characteristics reveals that ctDNA sequences are generally more fractionated and shorter in length than background DNA fragments in plasma [13].

**Figure 1:**
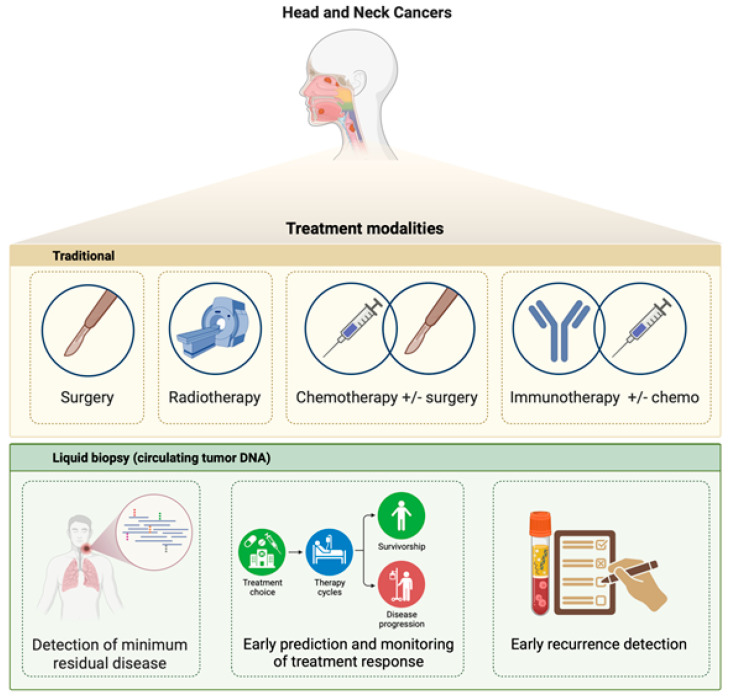
Overview of therapeutic strategies for head and neck cancers. Traditional modalities include surgery, radiotherapy, chemotherapy (with or without surgery or radiotherapy), and immunotherapy (with or without chemotherapy). Emerging liquid-biopsy approaches that analyze circulating tumor DNA (ctDNA) enable detection of minimal residual disease, real-time assessment of treatment response, and early identification of recurrence.

It is desirable that ctDNA analysis could replace tumor tissue profiling as it offers many advantages, such as being less invasive and dynamic and allowing the real-time monitoring of genomic and epigenetic alterations during the treatment course. As a result of increasing evidence for the usability of ctDNA in cancer management in the late 2000s, the term ‘liquid biopsy’ was first introduced as a new diagnostic concept in the 2010s to aid in the early detection of cancer, stratify patients for optimal treatments, detect minimal residual disease (MRD) (Figure 1), and thus predict recurrence after primary treatment in cancer patients [15,16,17]. In future, the ability to monitor the tumor DNA over time could make clinicians more flexible in making dynamic changes in patient management, which will improve the clinical outcomes of cancer patients.

Methods and technologies for ctDNA detection and analysis have made significant progress over time with a considerable improvement in their sensitivity [18]. ctDNA detection may be targeted or untargeted [19]. Targeted approaches are employed to search for known tumor-specific genetic alterations, such as driver mutations or genomic sequences of integrated cancer-related viruses, with the absolute quantification of target sequences. PCR-based techniques, such as quantitative PCR (qPCR), droplet digital PCR (ddPCR), and BEAMing, are the most commonly used methods for targeted ctDNA detection and quantification. NGS approaches allow the quantification of the proportion of mutated variant reads for a given mutation, also known as the variant allele frequency (VAF). VAF is the percentage of sequence reads representing a particular DNA variant divided by the total number of reads at that locus and indicates the proportion of mutated tumor DNA within plasma cfDNA.

Next-generation sequencing (NGS)-based sequencing technologies as untargeted approaches can detect undefined genetic and epigenetic alterations and produce results as ratios. Undefined genetic alterations may include unknown mutations, deletions, insertions, and chromosomal instabilities; the latter is quantified via the copy number instability (CNI) score of ctDNA.

A common approach to using ctDNA in oncology is to use tumor-informed ctDNA testing based on whole tumor exome sequencing (WES) or whole tumor genome sequencing (WGS) of tumor tissue to identify specific alterations in individual patients. This then enables the development of personalized assays to track plasma ctDNA during a treatment or follow-up phase [20]. The performance of ctDNA detection techniques concerning sensitivity may vary in different body fluids. Mattox et al. compared the performance of NGS, ddPCR, and qPCR in the quantification of HPV ctDNA in plasma samples and oral rinses from patients with HPV-positive oropharyngeal cancer [21]. In plasma, NGS and ddPCR showed similar performances, with sensitivities of 68.3% and 69.8%, respectively, and these rates were much higher than the sensitivity (20.6%) of qPCR. In oral rinses, NGS harbored considerably greater sensitivity (75%) compared to ddPCR (8.3%) and qPCR (2.1%), indicating the necessity of the adaptation of the technique to the fluids used.

## 3. Circulating Tumor DNA Analysis in the Management of HNSCC Patients

The detection and monitoring of disease recurrence or progression in patients with HNSCC can be performed by the clinical examination, imaging, or biopsy of suspicious lesions. These practices are, however, related to some limitations, including patient discomfort, time, and cost issues, and may have inherent inaccuracies in detecting recurrence [22]. As with other solid cancers, ctDNA holds great promise in HNCs, and the liquid profiling of ctDNA has shown a clinical utility in the detection, prognostication, and monitoring of patients with various forms of HNSCCs [22]. For instance, the study by Silvoniemi and colleagues has documented that a complex mutational landscape in cfDNA is positively correlated with metabolic tumor burden measured with FDG-PET imaging in HNSCC patients, and the combined use of ctDNA analysis and FDG-PET/CT may improve the predictive and prognostic evaluation of HNSCC tumors in the initial diagnosis and in the surveillance after definitive therapy [23].

TP53 mutations are the most frequent genetic alterations in HNSCC tumors, with a prevalence of up to 70%, albeit with variable frequency in different anatomical locations of HNC [24]. They can also be detected in plasma DNA with a high concordance with TP53 mutations in tumors [25]. The absolute quantification of TP53 mutations in plasma samples of six HNC patients by ddPCR revealed highly varying concentrations ranging from 2.2 to 422 mutational copies per ml of plasma [26]. TP53-based ctDNA was associated with worse progression-free survival (PFS) and regional metastases, indicating its beneficial role as a prognostic biomarker [27]. Wei et al. analyzed data from The Cancer Genome Atlas for mutated genes and found that TP53 is the most frequently mutated gene in HNSCC. Patients with a TP53 mutation in their tumors responded less well to immunotherapy but were highly sensitive to chemotherapy compared to patients without the mutation. They demonstrated that the detection of TP53 mutations in the bloodstream is feasible, as six of nine (66.7%) HNSCC patients had TP53 mutations detected in their tissue. This highlighted the potential for predicting treatment outcomes in HNSCC patients by detecting TP53 mutations in plasma DNA [28]. Studies using the targeted sequencing of driver genes in plasma DNA in recurrent and/or metastatic HNSCC have shown that ctDNA analysis has the potential to identify actionable mutations that may aid in treatment decisions in advanced HNSCC [25,29,30].

A further explorative study by Payne et al. evaluated the utility of ctDNA in detecting intra-tumoral heterogeneity (ITH) in HNSCC, as spatial and temporal ITH may affect ctDNA analysis. In a small cohort of treatment-naive HNSCC patients with advanced (III/IV) stage disease *n* = 9, mostly oral cavity tumors), DNA from two intra-tumoral sites (tumor core and margin) was used for targeted exome sequencing of a nine-gene panel in plasma samples collected at baseline and at selected post-treatment time points. The results showed that genomic ITH was high among the patients, with 86.9% heterogeneity, meaning the variants were detectable in one tumor site only (core or margin). Across all the patients, ctDNA identified a mean of 12.9% of all the tumor-specific variants of which 55.6% were specific to a single tumor subsite only. Also, serial post-treatment ctDNA analyses were capable of predicting recurrence ahead of the clinical detection of treatment failure in two patients who recurred [31]. In addition to somatic variants, the same group also demonstrated the usability of cfDNA methylation for ITH assessment in the same cohort of HNSCC patients [32].

In addition to mutational ctDNA analysis, quantifying plasma DNA was also shown to be useful in the management of HNSCC patients. Koukourakis et al. measured plasma DNA levels by using a Qubit fluorometer in HNSCC patients (*n* = 47) with locally advanced diseases and looked at its role in predicting the treatment response following definitive CRT. The authors found that a high pre-/post-CRT cfDNA ratio indicates incomplete tumor eradication and poor survival. Importantly, this information is available earlier than routine image-based scanning and may be a useful aid in stratifying HNSCC patients at a high risk of local and distant recurrence [33].

In this review, we provided an update on research on the role of ctDNA in the management of HNSCC patients and included further citations with full-text content available (Table 1) [34,35,36,37,38,39,40,41,42,43,44,45,46,47,48,49,50]. Most studies aimed to assess the promise of ctDNA in predicting the treatment response, the detection of MRD, and the risk of recurrence after treatment. We structured the article according to the treatment modality used: (i) Studies with surgery only. (ii) Studies with RT only. (iii) Studies with multiple treatment modalities (e.g., surgery plus RT or CRT, or CRT alone). (iv) Studies with immunotherapy. Since there are a sufficient number of review articles and/or meta-analyses on the impact of circulating HPV-DNA and EBV-DNA in the detection, treatment monitoring, and the surveillance of patients with virus-related HNSCC [51,52,53,54,55,56], here we focused on the role of non-viral ctDNA in the management of HNSCC patients. The inclusion of current immunotherapy studies in the review and the reviewing of studies by the treatment modality represents the main novelty of this article over other published review articles.

### 3.1. Studies with Surgery Only

ctDNA is increasingly being incorporated into various stages of cancer management [57]. One of these applications is its use for the detection of MRD, which reflects the incomplete eradication of cancer cells after the completion of primary treatment. The assessment of the treatment response relies on radiologic imaging, which is, however, unable to detect MRD. ctDNA positivity in the post-treatment analysis is associated with a higher risk of recurrence and worse survival in multiple cancer types [58]. Research conducted shows that in HPV- and EBV-related HNSCCs, such as oropharyngeal carcinoma and nasopharyngeal carcinoma, circulating HPV- and EBV-DNA measured at treatment completion are useful in predicting recurrence, but not for virus-negative HNSCC [59,60]. This has driven research on the impact of non-viral ctDNA on the prognostication of HNSCC. The study by Flach S et al. investigated the potential of ctDNA in detecting MRD and disease recurrence in patients with p16-negative resectable HNSCC who underwent curative-intent primary surgical resection. The locations of the tumors were the oral cavity (*n* = 5), oropharynx (*n* = 2), larynx (*n* = 7), hypopharynx (*n* = 4), and secondary primary tumor (*n* = 1). DNA from formalin-fixed, paraffin-embedded (FFPE) tumor tissue was subjected to exome sequencing to generate a patient-specific panel of targets (corresponding to 48 primer pairs), enabling the detection of at least one somatic variant in plasma. Serial plasma samples (total 103) were available at baseline (1–4 days before resection) and postoperatively (2–7 days after resection). To detect tumor-specific variants in serial pre- and postoperative plasma samples as evidence of MRD and recurrence, a sequencing-based personalized assay (RaDaR^TM^) was utilized. In 17 patients analyzed, the number of somatic variants ranged from 34 to 52, and tumor-specific variants were detected in all the pre-operative plasma samples (100% sensitivity). In the post-surgery samples, ctDNA could be detected at levels as low as 0.0006% VAF. In five patients who relapsed, ctDNA was detected before progression (lead-times 108 to 253 days). The authors concluded that ctDNA as a biomarker harbors the potential for detecting MRD and recurrence in HNSCC, underscoring the feasibility of personalized ctDNA assays for detecting clinical recurrence [34].

A more recent study by Marret et al. [35] also aimed to evaluate the clinical utility of ctDNA in MRD detection in HNSCC patients with nonmetastatic, resectable disease. The study included 41 patients with tumors in the oral cavity (*n* = 12), oropharynx (*n* = 15), larynx (*n* = 3), and hypopharynx (*n* = 1). Most of them were HPV-negative. They were treated with surgical intervention with curative intent as a part of a clinical trial (SCANDARE study). During the follow-up (median 41 months), disease recurrence occurred in thirty-one patients (76%). Targeted NGS on tumor tissues was performed using a custom panel of 571 genes covering frequently mutated genes in HNSCC. ctDNA detection was also based on the targeted sequencing of plasma DNA to detect somatic variants identified in tumor tissues to compile a patient-specific panel with a median of 14 variants. Furthermore, this panel was combined with a fixed core panel of 40 genes, covering a list of frequently mutated genes in HNSCC, to detect mutations in serial plasma samples. Availability of plasma samples was 100% at surgery, 83% (34/41) within 14 weeks after surgery, 44% at six months (18/41), and 71% at the detection of recurrence (22/31). The sensitivity of ctDNA detection at surgery was 51% (21/41 patients), 44% (15/34) within 14 weeks after surgery, and 68% at recurrence (15/22 patients). ctDNA-positive plasma samples at surgery had a lower prevalence of mutated *TP53* and *FAT1* than in tumors, while *CDKN2A* and *PTEN* mutation rates were similar. During longitudinal plasma sampling, newly emerging mutations (not present in tumors) were reported in 9/21 patients (43%). ctDNA-based MRD detection predicted clinical recurrence with a median lead-time of 9.9 months (range 8.0–14.5 months) in 17 out of 27 patients (63% sensitivity at levels as low as 0.1% VAF). ctDNA in samples obtained within 14 weeks after surgery correlated with disease recurrence after adjusting for classical prognostic variables (HR 3.0; *p* = 0.03). This study again reveals the usefulness of ctDNA for MRD detection in resectable HNSCC patients.

### 3.2. Studies with Radiotherapy Only

In recent decades, advances in RT techniques have expanded their indications in clinical practice as approximately 50% of cancer patients, particularly those with HNC, lung cancer, early breast cancer, and prostate cancer, undergo RT in any phase of their treatment [61]. Advanced forms of conformal RT, such as intensity-modulated RT (IMRT) and image-guided RT ensure the delivery of high doses of radiation to target volumes with minimized exposure to normal tissues. Such techniques are employed in precision radiation oncology based on individual dosing based on radiation sensitivity, which in turn requires biomarkers that can easily and reliably predict the RT response. In this context, Cho WK and colleagues interrogated the feasibility of cfDNA in monitoring the response to RT in solid cancers, including oropharynx, lung, and esophageal cancers. In 20 patients out of 23, RT was applied in the range of 30–68.4 Gy and with a curative intent (10 salvage, 6 neoadjuvant, and 4 definitive), while it was palliative in the remaining three. The response to RT was evaluated according to the Response Evaluation Criteria in Solid Tumors (RECIST) version 1.1. Serial cfDNA monitoring was performed before RT, one week after RT, and one month after RT in 23 patients. The ctDNA analysis was based on a low-pass, whole-genome sequencing (WGS) strategy and led to the calculation of the I-score, which reflected the overall copy number instability as an index of the MRD. The baseline I-score significantly correlated with the gross tumor volume (Spearman rho = 0.419, *p* = 0.047). At one month after RT, complete response and partial response were detected in 2 and 13 patients, respectively, whereas 5 patients had stable disease, and 3 others experienced disease progression. The I-score one month after RT (e.g., 4.79) was significantly lower than that at baseline (5.27, *p* = 0.002), indicating a reduced tumor burden. The findings of this study demonstrate the feasibility of ctDNA analysis based on copy number instability to MRD after RT in solid cancers [36].

In general, recurrent and/or metastatic HNSCC is an incurable disease, and the prognosis of these patients is very poor, with a median overall survival of up to 15 months [62]. However, high-precision RT can give us an opportunity to cure recurrent HNCs, particularly recurrent nasopharyngeal carcinomas, and even oligometastatic carcinomas. Thus, early recurrence detection in HNSCC patients is important for improving their prognosis. Locoregional recurrence after RT is common in locally advanced HNSSC patients, and therapeutic options are limited for these patients. Curative-intent resection as surgical salvage is the preferred option for patients with low-volume tumors [63]. As complete resection is not possible in the majority of these patients, re-irradiation represents a frequently-used option in patients with locally and/or regionally recurrent HNC without distant metastatic disease [64]. IMRT is widely used for the re-irradiation of locally recurrent HNCs. Re-irradiation (alone or in combination with CTx) can improve local disease control and survival rates [65]. However, although re-irradiation for recurrent HNC is clinically beneficial, treatment-related side effects and early tumor progression are often a concern [66], and predictive biomarkers are needed to tailor the treatment intensity to the patient’s risk. Thus, Janke and colleagues intended to correlate serial ctDNA analysis with the disease outcome after re-irradiation in locally advanced recurrent HNSCC. As part of a phase II randomized clinical trial, patients were either exposed to carbon ion re-irradiation or volumetric modulated arc therapy, with total doses ranging from 51 and 60 Gy delivered in six fractions per week. Plasma samples (*n* = 94) from 16 locally advanced HNSCC patients (two oral cavity, three oropharynx, three nasal cavity, three nasopharynx, two hypopharynx, two sinuses, and one skull base) and 57 healthy donors were collected before re-iradiation, after 5 and 10 RT fractions, at RT end, and at routine follow-up points. The ctDNA analysis was based on the estimation of copy number variations by low-coverage WGS. For each plasma sample, based on the copy number variation profile, a copy abnormality (CPA) score was calculated to quantitatively assess the extent of chromosomal instability detectable in the plasma. Copy number alterations in plasma DNA were detected in 8/16 patients and 35 out of 94 plasma samples (37% sensitivity). ctDNA detection was performed after 5 and 10 fractions (both: log-rank, *p* = 0.050) and at the end of RT correlated with PFS (log-rank, *p* = 0.006). In patients with shorter PFS, ctDNA levels reduced from the re-irradiation baseline and from the five-fraction time point to the end of re-irradiation (log-rank, *p* = 0.005). ctCPA score increases between re-irradiation end and disease progression were observed in seven out of eight patients, highlighting the relevance of tracking disease recurrence by copy number instability in the plasma. Notably, in patients, molecular progression (e.g., rising ctCPA scores) was detected before imaging-based tumor progression. The findings of this study emphasize that quantifying ctDNA during re-irradiation may be useful for monitoring the therapy response and the personalization of treatment and dosing [37].

### 3.3. Studies with Multimodal Treatment Modality

Lele et al. asked whether ctDNA could be beneficial as an adjunct method to overcome the limitations of PET and select HNSCC patients with a higher risk of recurrence. Twenty-nine patients receiving definitive treatment were recruited. The patients had an oral cavity (*n* = 6), laryngeal (*n* = 12), oropharyngeal (*n* = 10), and hypopharyngeal cancer (*n* = 1). Advanced-stage patients made up the majority of the cohort. The majority of the patients (*n* = 21, 72%) had cT3/cT4 stage disease, and 62% (*n* = 18) of the patients had positive nodal disease. Primary treatment was surgery for 10 patients (34%) and definitive CRT for 19 (66%). All the patients with surgery as their primary treatment received platinum-based adjuvant CRT. The median follow-up was 16 months (6 to 43 months). ctDNA was detected by a multiplex PCR NGS assay, and ctDNA sampling was performed at different time points (1 to 3, 6, 9, and 12 months after definitive treatment). Seven patients with a biopsy-proven recurrence had post-treatment ctDNA detected. Six had positive results at the first follow-up, and one became positive by 6 months post-treatment. The most frequently detected mutations were TP53, CDKN2a, and PIK3CA genes. ctDNA positivity after definitive treatment was associated with a higher risk of disease recurrence (HR 9.94, *p* = 0.015) as it was possible to identify recurrence via ctDNA with 100% specificity at a sensitivity rate of 78%. In contrast, the performance of PET in detecting recurrence was lower, with a sensitivity of 78% and a specificity of 68%. The authors concluded that ctDNA can act as an excellent complementary test to post-treatment PET and can help physicians make decisions in cases with inconclusive PET results [38].

Recent advancements in RT techniques make it increasingly feasible to safely deliver high, ablative RT doses with local curative intent and improve disease outcomes in cancer patients. Balázs et al. aimed to assess the value of ctDNA profiling in the longitudinal monitoring of high-dose RT in a heterogeneous cohort including patients with locally advanced HNSCC (two oropharynx, two tonsillar carcinoma, two base of tongue, and one hypopharynx) and oligometastatic disease (defined as having 1–5 metastases) with various primary tumors. Healthy subjects and patients with polymetastatic lesions (defined as having more than five metastases) served as control samples. HNSCC patients received CRT with 70 Gy irradiation and cisplatin over 7 weeks, and patients with oligometastatic disease were treated with stereotactic ablative treatment of 35–50 Gy in five fractions. Plasma samples were collected before, during, and after one week of treatment, on the last day of treatment, and during follow-up after 3, 6, and 12 months. In a tumor-agnostic approach, cfDNA from 93 samples was sequenced by low-pass WGS, and copy number alterations and fragment length distributions were correlated to clinical and imaging findings. The authors have shown that tumor-specific copy number variants can be identified above a tumor fraction threshold of 2%. Copy number alterations were detected in four out of seven polymetastatic cancer patients (57% sensitivity at 100% specificity), 1/7 in oligometastatic, and 1/7 in HNSCC patients (14% sensitivity at 100% specificity), and correlated with radiological progression following RT in these patients. A strong correlation between tumor-specific fragment length features and copy number-based tumor fraction estimates was also noteworthy. Despite the small sample size, this explorative study, performed in a heterogeneous cohort, indicates that an increased tumor fraction measured via copy number and fragment length analysis in plasma is associated with systemic tumor load and aggressiveness, and this approach can be adapted to treatment strategies [39].

Assessing cfDNA kinetics in the plasma of patients with HNSCC before and after therapy can provide useful information regarding locoregional control and the likelihood of recurrence. Several studies evaluated the utility of ctDNA in predicting recurrence in locoregionally confined/locally advanced HNSCC treated with surgery plus RT/CRT or CRT. Burgener et al. performed a tumor-naive ctDNA analysis in patients with HPV-negative, locoregionally confined HNSCC by the simultaneous profiling of mutations and methylation. All the patients (stage I-IVA) enrolled in the study were treated with surgery (*n* = 30) plus RT (*n* = 11) or CRT (*n* = 15). A mutational analysis was performed by the CAncer Personalized Profiling by deep Sequencing (CAPP-seq) approach, and the methylation analysis was done by the sequencing of methylation-based, immunoprecipitated plasma DNA (cfMeDIP-seq). Plasma samples (total 77) were collected at baseline, during, and upon the completion of treatment. Mutations were detected in 20/30 patients (66% sensitivity) at baseline with a VAF ranging from 0.14% to 4.83. A differential methylation analysis identified 941 hypermethylated regions enriched with HNSCC-specific methylation patterns. Mutation- or methylation-containing plasma DNA fragments were shorter than fragments in the background DNA. ctDNA (mutation or methylation) positivity at the pretreatment phase was associated with poor OS (HR = 7.5; *p* = 0.025). Various ctDNA kinetics during therapy were determined by tracking methylation changes in plasma. At the last time point of sampling, some of the patients had complete clearance, while a reduction of ctDNA of >90% was defined as partial clearance, and an increase or reduction of ≤90% was defined as no clearance. Notably, the patients (5 out of 18) without ctDNA clearance were more likely to suffer from disease recurrence than those with complete or partial clearance (HR = 8.73, log-rank *p* = 0.0046). Interestingly, all the patients with higher ctDNA levels at the last point of sample collection than at baseline experienced disease recurrence. The findings of this study confirm the potential clinical use of tumor-naive ctDNA detection in the management of HNSCC patients [40].

Orland et al. asked whether ctDNA can be used as a measure of MRD following surgical intervention in HNSCC patients. The study enrolled 28 patients who underwent the surgical removal of primary laryngeal or oral cavity cancer. The majority of patients (*n* = 18) were stage IV, while five were stage III, three were stage II, and two were stage I. Of 28 patients, 19 were classified as having high-risk cancer as they had extranodal disease or positive margins during surgery, and 15 of them (15/19) received adjuvant CRT. The ctDNA analysis was performed using a personalized commercial ctDNA test (Signatera). Of the high-risk patients, 11 out of 18 with data available had positive ctDNA before adjuvant therapy (61% sensitivity). In contrast, a small portion of the patients without high-risk features had positive MRD postoperatively and experienced disease relapse. In the patients without high-risk features, the rate of disease recurrence was lower than in high-risk patients (22.9% vs. 54.5%, respectively), which correlated with ctDNA. This study shows that the detection of ctDNA postoperatively is common in HNSCC patients with high-risk features and that it may help modulate and intensify treatment in high-risk patients [41].

Chikuie et al. were also interested in evaluating the clinical utility of ctDNA in predicting recurrence in patients with HNSCC. Of 20 patients with HNSCC recruited in the study, eight (40%) had stage I disease, one stage II, six stage III, and five stage IV. The majority of the cases (*n* = 16) were oropharyngeal cancer, and 11 of them were p16-positive. One patient had oral cancer, and three hypopharyngeal cancer. The primary treatment was surgical resection for nine patients, while the remaining ones were treated with chemoradiotherapy. Multiple plasma samples (*n* = 55) were collected before and after treatment. Somatic mutations in 71 oncogenes and copy number alterations with a detection rate of >5% in HNCs, according to the International Cancer Genome Consortium data portal, were searched by exome sequencing. ctDNA before treatment was detected in 10 cases (50% sensitivity) and was associated with tumor staging and recurrence. During the surveillance period, no ctDNA was detected in 13 recurrence-free cases, but it was detected in five of the seven cases with disease recurrence, corresponding to a 71% sensitivity of ctDNA detection in recurrent cases. Furthermore, the post-treatment, relapse-free survival time was significantly shorter in the cases with ctDNA detection (9.6 ± 9.1 months) than in those without detected ctDNA (20.6 ± 7.7 months, *p* < 0.01). Notably, in two recurrent patients with ctDNA detected after treatment, ctDNA detection was more sensitive in predicting recurrence than computed tomography-based imaging. The findings of this study provide further evidence that ctDNA profiling during follow-up is useful in patients with HNSCC for predicting treatment response and recurrence [42].

A further study that was interested in the predictive capacity of liquid profiling during CRT in patients with locally advanced HNSCC was conducted by Hilke and colleagues [43]. Twenty patients with cancer in the oropharynx (*n* = 14), hypopharynx (*n* = 4), or oral cavity (*n* = 2) were recruited in this study. The patients were treated with definitive CRT, with cumulative radiation doses of 70–77 Gy by IMRT and concomitant CTx with cisplatin weekly or combination therapy of 5-fluorouracil and mitomycin C. The response to treatment was evaluated by clinical investigations and tomography scans. For longitudinal ctDNA analysis, a total of 99 blood samples were collected at baseline, during therapy (three samplings), and after CRT. The patients were followed-up for up to 1168 days (median 823 days). Ten patients experienced local or distant relapses, with most relapses occurring in the first year. Of several hundred mutations detected in FFPE tumor tissues, 127 were evaluated as driver mutations, and their genomic regions were sequenced from plasma DNA. At a VAF of greater or equal to 0.05%, 85% of the patients (17/20, 85% sensitivity) had detectable levels of driver mutations at baseline in their plasma, which correlated significantly with the gross tumor volume (*p* = 0.032). The genes in the TP53-, NOTCH-, HIPPO-, and PI3K-pathways or the chromatin modifiers were frequently mutated. The tumor allele fraction of ctDNA was negatively correlated with the treatment course (*p* < 0.05). In the patients with ctDNA detection at the first follow-up point, disease recurrence occurred. All of the patients with detectable MRD had a relapse. The findings of the study show again that ctDNA is an informative biomarker of disease recurrence in HNSCC patients.

The study by Sanz-Garcia aimed to investigate the impact of ctDNA analysis in MRD detection in locally advanced HNSCC patients who had stage III (HPV-positive) and stage III-IVB (HPV-negative) disease. Despite aggressive treatment, the prognosis is poor in these patients, with a substantial risk of distant metastases [67,68]. Tumors were located in the oropharynx (*n* = 23), larynx (*n* = 5), oral cavity (*n* = 3), and hypopharynx (*n* = 1). The patients received curative intent treatment: surgery followed by adjuvant CRT, definitive RT, or CRT, where CRT was the most frequent (78%) treatment. Serial plasma samples (86 in total) were collected before treatment and 4–6 weeks (P1) and 8–12 weeks (P2) after treatment. All the patients underwent a radiological assessment at the sampling point P2. The ctDNA analysis was performed by two approaches: using the tumor-informed RaDaR^®^ assay and a tumor-naive assay (CAPP-seq), including the sequencing of a panel of genes optimized for HNSCC. With the RaDaR assay, ctDNA was detected in 15 of 17 samples (88% sensitivity) at baseline and was not associated with RFS. No patients had radiological residual disease at P2. Seven clinical relapses occurred during the follow-up period (median 25 months). In two patients who relapsed within the first year of definitive therapy, ctDNA showed MRD at P2 using RaDaR. ctDNA detection during follow-up was associated with shorter RFS (*p* < 0.001). RaDaR assay was superior to CAPP-seq for MRD detection with sensitivity and specificity rates of 40% and 100% vs. 20% and 90.5%. The results showed that the RaDaR assay-based MRD detection in follow-up plasma samples may identify HNSCC patients who relapse within 1 year [44].

Another study that addressed the question of whether personalized ctDNA analysis could be helpful in monitoring treatment response and recurrence in HNSCC patients was conducted by Kogo et al. [45]. These patients had cancers in the oral cavity (*n* = 5), oropharynx (*n* = 5), hypopharynx (*n* = 3), larynx (*n* = 7), and the external auditory canal (*n* = 6). The majority of the patients (65.4%) had stage IV disease, and most of the patients were categorized as having advanced disease. One patient was treated with RT, nine with surgery, five with CRT, three with induction CTx and CRT, two with induction CTx and surgery, two with surgery plus CRT, and three with induction CTx, surgery, and CRT. The patients were followed-up for a median average of 563 days. At a VAF rate of >10%, 31 genes frequently mutated in HNSCC were identified as candidates for ctDNA analysis after sequencing tumor and peripheral blood mononuclear cell DNA in 26 patients. ctDNA monitoring was performed by dPCR. The most frequent mutations (58.2%) were detected in *TP53* in tumor samples. For technical reasons, longitudinal ctDNA monitoring in plasma was performed in a total of 18 cases, and in two cases (2/18), ctDNA was not detectable during the follow-up period (88% sensitivity of ctDNA detection in the monitoring phase). Seven patients who relapsed became ctDNA positive or did not become negative after curative treatment. The patients (*n* = 11) who remained ctDNA-negative after the initial curative treatment were alive without recurrence and had a better prognosis than those who became ctDNA-positive (*p* < 0.0001). The authors conclude that individualized ctDNA monitoring based on patient-specific mutations may be a promising biomarker for detecting recurrence in HNSCC patients to enable the earlier initiation of salvage therapy.

A retrospective study by Hanna et al. evaluated ctDNA detectability in locally advanced, predominantly HPV-negative (68%) HNSCC and the related post-treatment ctDNA changes with disease outcomes. Sixty-five percent of the patients (75/116) were former or current smokers. The oral cavity (47%), larynx/hypopharynx (22%), and oropharynx (16%) were the most frequent tumor sites. At the initial staging, most of the patients (60%) had T0– disease, 92% were N0–2, while a subset (17%) had distant metastases. Fifty-three percent were treated with definitive concurrent CRT as initial therapy. The patients with locoregionally advanced, unresectable, or distant metastatic disease (18%) received upfront systemic treatment. A tumor-informed ctDNA analysis was performed using a personalized, commercial ctDNA assay (Signatera), which utilizes 16-plex PCR from matched tumor and blood samples. The tissue mutational analysis was successful in 86% (100/116) of the patients. Of these, 75% had detectable ctDNA at baseline, which ranged in a broad interval from 0.03–4050 mean molecules per mL of plasma. The multivariate analysis revealed that ctDNA detectability or levels were not associated with any clinical features in the pretreatment phase. During the follow-up (median 5.1 months), 55% of patients had >1 test result (total 194 samples). Of 55 patients, 31% remained ctDNA positive following treatment initiation. ctDNA positivity at the post-treatment stage was significantly associated with worse PFS (HR, 7.33; *p* < 0.001). One-year OS rates were 89.1% vs. 100% in the ctDNA-positive and -negative patients, respectively (HR, 7.46; *p* = 0.155). The results of this study confirm the impact of ctDNA positivity as an indicator of disease progression and inferior survival [46].

Honore et al. used a tumor-agnostic approach to evaluate the utility of ctDNA to detect MRD in unselected, locally advanced HNSSC patients to predict PFS and OS. Fifty-three patients with plasma samples available at the pretreatment and post-treatment stages were enrolled in the ctDNA profiling. The oropharynx (*n* = 29, 54.7%) was the most frequent tumor site. Forty-nine patients (92.4%) received curative-intent CRT as the primary treatment, while the remaining four patients (7.6%) were treated with surgery. The ctDNA analysis was based on the sequencing of a panel of 26 genes frequently mutated in HNSCC and two HPV-16 genes. The VAF ranged from 0.2% to 20.2% (median 0.87%) for ctDNA. In patients with positive cDNA at baseline, MRD was defined as the detection of ctDNA at any time point within 1–12 weeks of the completion of curative treatment. In the pretreatment samples, the ctDNA positivity rate was 77% (41/53). Of the 41 patients with positive ctDNA at baseline, 17 were MRD-positive (41% sensitivity) after treatment, with a median VAF of 0.29%. With a median follow-up of 31 months, the PFS rate at 2 years was 60.3% (median 34.7 months) and OS was 78% (median not reached) in the unselected patients. The post-treatment ctDNA presence in plasma was associated with outcomes, as the median 2-year PFS rate was 23.5% and 86.6% in the MRD-positive and -negative patients, respectively (*p* < 0.05). Importantly, the median OS was 28.4 months for MRD-positive patients and was not reached at the time of analysis for the MRD-negative cohort (*p* = 0.011) [69].

Koukourakis and colleagues conducted a study in patients with inoperable HNSCC in which they aimed to explore the feasibility of ctDNA in identifying high-risk patients for locoregional recurrence or metastatic progression. Primary tumors of 38 patients (17 larynx, 3 oropharynx, 5 oral cavity, 3 hyphopharynx, 6 nasopharynx, 2 parotoid gland, 2 neck) were exposed to a total of 56–59 Gy radiation in 22 fractions using image-guided RT and a volumetric modulated arc therapy. The patients also received concurrent CTx with cisplatin (35–40 mg/m^2^ per week), or cetuximab (250 mg/m^2^/week), or their combination. Blood samples were collected pretreatment and after the completion of CRT, and the patients were followed-up for up to 36 months (median 15 months). The ctDNA analysis was based on a commercial sequencing assay of a panel of genes (approx. 70) frequently mutated in HNC to detect point mutations and other alterations, such as deletions, insertions, fusions, and CNVs. The limit-of-detection of point mutations ranged from 0.1 to 1.3 (median 0.4) and from 0.1 to 1.2 (median 0.5), before and after, respectively. With a mutation rate of 26.3%, *TP53* was the most commonly mutated gene in the pretreatment samples. TP53 mutations persisted in 4 out of 10 patients after CRT, and seven additional patients who were negative for *ΤP53* mutations at baseline turned positive after CRT. Mutations in genes *EGFR*, *AR*, *FGFR3*, and *FBXW3* were detected in the pretreatment plasma samples of four patients. After the end of CRT, the patients with a response (complete or partial) to CRT had fewer mutations than in nonresponders (26.6% vs. 75%, respectively; *p* = 0.03). The patients with ctDNA positivity before and after CRT had poorer locoregional-free survival (LRFS) (*p* = 0.02) and distant-metastasis-free survival (*p* = 0.04). The findings of this study show that ctDNA profiling before and after CRT could help clinicians identify patients with a high risk of locoregional recurrence [48].

### 3.4. Studies Including Immunotherapy

Immune checkpoint blockade (ICB) therapy is based on blocking inhibitory signals of T cell activation to stimulate anti-tumor immune responses. Since its successful introduction as a treatment modality for advanced melanoma in 2011, ICB using the inhibitors of PD1 or PD-L1 has become a standard of care in cancer treatment providing long-term clinical benefits in many patients with multiple tumor types and even inducing a durable response in a subset of patients [70]. Following the clinical trials CheckMate141, KEYNOTE-012, and KEYNOTE-048, ICB based on the PD1 inhibitors pembrolizumab and nivolumab has also become the standard in the treatment of recurrent/metastatic HNSCC, which is associated with a poor prognosis and a median survival of 10–15 months [71]. However, only a small portion of patients (approx. 20%) benefit from ICB therapy. Thus, the discovery of predictive biomarkers is critical to optimizing treatment strategies and improving the survival of HNSCC patients. Wilson et al. were the first to report that ctDNA predicts poor survival after immunotherapy [25], demonstrating the potential of liquid profiling in HNSSC management following immunotherapy. In a clinical trial demonstrating the efficacy of sitravatinib, a tyrosine kinase inhibitor, plus nivolumab in locally advanced oral cancer (10 patients) in the neoadjuvant setting, a relative decrease in ctDNA levels and an increase in cfDNA levels days before surgery compared to baseline correlated with tumor downstaging by the effect of sitravatinib and nivolumab [72]. Also in other solid tumors, ctDNA proved to be a useful biomarker for the real-time assessment of the disease burden under ICB therapy and for predicting treatment efficacy of cancer immunotherapy [73,74,75].

Three further studies utilized plasma ctDNA for the monitoring of ICB in recurrent/metastatic HNSCC. Honore and colleagues aimed to assess the predictive impact of ctDNA profiling on the efficacy of a single-agent PD1 inhibitor (pembrolizumab or nivolumab) in recurrent/metastatic HNSCC. Of recurrent/metastatic HNSCC patients treated with immunotherapy enrolled in a biomarker trial, 44 met the inclusion criteria for this study. The majority of the patients had cancers of the oral cavity (*n* = 13) and the oropharynx (*n* = 17). The remaining tumors were hypopharynx (*n* = 4), larynx (*n* = 3), HNC unknown primary (*n* = 6), and two locations (oropharynx and oral cavity, *n* = 1). The ctDNA analysis was performed by a tumor-agnostic sequencing assay that included 37 genes frequently mutated in recurrent/metastatic HNSCC and two HPV16 genes. The assessment of the concordance between ctDNA kinetics (ΔctDNA) and the overall response according to RECISTv1.1 evaluation was the main goal of the study. ΔctDNA was defined as the difference in the mean VAF between the plasma sample collected 6–10 weeks after the start of ICB therapy and the pretreatment sample. In 80% of the 44 patients, ctDNA was detectable in pretreatment samples, with the genes *TP53*, *SPEN*, *EP300*, *SMG1*, and *KMT2C* being the most frequently mutated. Among the HPV16-negative patients (*n* = 41), the sensitivity of ctDNA detection was 78% in the pretreatment samples. In the overall cohort, three complete responses and six partial responses were observed (the overall objective response rate is 20%). Among the 35 patients with ctDNA positivity at baseline, 17 had a decline (negative ΔctDNA) in the mean VAF, and the majority of them (76.4%) achieved either a complete response, partial response, or stable disease, with four cases experiencing disease progression. Among those with an increase (positive ΔctDNA) in the mean VAF, the majority of the cases (72%) had progressive disease. The concordance between ΔctDNA and the imaging response according to RECISTv1.1 was observed in 74% of cases. The median PFS was significantly higher (8.6 months) in the negative ΔctDNA group than in the positive ΔctDNA group (*p* = 0.057). Similarly, the median OS was 18.1 and 8.2 months in both groups, respectively (*p* = 0.13). The results of this study reveal that ctDNA kinetics provide promising information in predicting the efficacy of PD1 inhibitors in recurrent/metastatic HNSCC [48].

In a further study, Noji et al. also prospectively evaluated the clinical use of ctDNA as a real-time biomarker for monitoring the treatment response in recurrent/metastatic HNSCC patients receiving immunotherapy. The study was part of the SHIZUKU-HN initiative and enrolled 10 HNSCC patients with 27 serial plasma samples available. All the patients had tumors in the oral cavity (the tongue, buccal mucosa, gingiva, and other oral locations). The ICB therapy with nivolumab was second-line therapy for three patients who were refractory to platinum-based CTx, whereas the other seven patients received pembrolizumab as first-line therapy. The primary endpoint of the study was to investigate the correlation between changes in the VAF and the radiological tumor response according to the RECIST 1.1 criteria. Plasma sampling was conducted at baseline, before ICB infusion, and at three subsequent time points (4 weeks, 6 months, and at progression). The patients were followed-up for a mean duration of 65 days (range: 28–273 days) from the first ctDNA sample collection, with radiological evaluations conducted within 0–1.6 months of each ctDNA collection time point. The ctDNA analysis was performed using the Guardant360 assay, which includes the targeted sequencing of 74 genes. Single-nucleotide variants, gene amplifications, fusions, short insertions or deletions, and splice variants were searched. The most frequently detected mutated gene in plasma DNA was *TP53*, with a detection rate of 44%, followed by the TERT promoter, *PIK3CA*, *GNAS*, and *CDKN2A*, with detection frequencies under 10%. The mean VAF significantly correlated with the tumor volume (Spearman’s ρ = 0.70, *p* = 0.001). Changes in the mean VAF, detected in the early phase of the treatment course, often preceded radiological progression. Among the patients with an achieved partial response or stable disease, the mean VAF remained relatively stable, whereas in the patients with progressive disease, early increases in the mean VAF occurred and accurately predicted disease progression. Even if *TP53* was the most frequently mutated gene, *BRAF* and *APC* mutations were more informative for the response in patients with partial response. This study further demonstrates the potential of ctDNA as a real-time biomarker for assessing the disease status and therapeutic efficacy of personalized ICB therapy [49].

The study by Taylor et al. included 55 metastatic HNSCC patients with tumors in the oral cavity (*n* = 13), oropharynx (*n* = 26), larynx (*n* = 8), hypopharynx (*n* = 5), and nasal cavity (*n* = 1). The number of metastases was 1–2 in 22 patients and 3 or more in 11 cases. Ninety-six percent of the patients included in the ctDNA analysis received one or more cycles of treatment, which included either first-line platinum-based systemic treatment (*n* = 16), second-line anti-PD1/PD-L1 monotherapy (*n* = 30), or a combination immunotherapy using two ICB agents (*n* = 7) in second-line or beyond settings. The median follow-up time was 8.2 months (1.8–38.2 months). Serial ctDNA sampling was done with plasma samples collected at baseline, before cycles 2 and 3, and at the time of disease progression (corresponding to time points T1–4). The ctDNA analysis was performed by sequencing a panel of genes (CAPP-seq) optimized for HNSCC. Besides ctDNA, the neutrophil-to-lymphocyte ratio (NLR) and platelet-to-lymphocyte ratio (PLR) as inflammation markers were also evaluated. At baseline, ctDNA was detected in 50 out of 53 (94.3% sensitivity) patients, with a median VAF of 4.3% (range 0.3–21.8%), and did not correlate with OS or PFS. Seven patients (five receiving single-agent ICB as the second line and two receiving CTx as the first line) experienced a partial response with an objective response rate (complete or partial) of 13%. Forty percent of the patients had a clinical benefit (defined as a complete or partial response, or stable disease). The median PFS and OS were 2.8 months and 8.2 months, respectively. Even if baseline ctDNA levels did not correlate with survival, a change in the ctDNA abundance from baseline to after one cycle of treatment (ΔVAF) predicted the improvement in PFS (*p* < 0.01) and OS (*p* < 0.01) in 49 patients. The patients who experienced a decrease in the ctDNA abundance from cycles 2 to 3 had longer OS (8.2 months) compared to those who had an increase in ctDNA (4.6 months) (HR 0.44, *p* = 0.03). In the multivariable analysis with 49 patients with a ctDNA VAF change from cycles 2 to 3, ctDNA ∆VAF retained an association with OS, indicating that early dynamic changes in ctDNA levels predicted both OS and PFS in metastatic HNSCC patients on systemic therapy [50].

## 4. Concluding Remarks

Despite intensified treatments, many HNSCC patients still have a high risk of recurrence. Differentiating residual disease from recurrent tumors after postoperative and radiation-induced changes by post-treatment biopsies is a difficult task. Furthermore, heterogeneity at different levels (e.g., intra- and intertumoral heterogeneity and the tumor microenvironment heterogeneity of HNSCC tumors) makes it impossible to cover all genomic and transcriptomic alterations in tiny tissue biopsies. Cancer-derived circulating biomarkers detected and quantified by highly sensitive and specific technologies may help detect MRD and predict recurrence in these patients post-definitive treatment, such as surgery or CRT. Active research on circulating biomarkers over the past two decades has revealed that ctDNA, the most prominent fluid molecular biomarker, has potential applications for treatment monitoring. It indicates genomic and epigenomic changes in tumors that can be tracked in real-time by serial sampling during and after therapy. In this review, we focused on non-viral ctDNA analysis in the management of HNSCC patients and categorized the studies according to the treatment modality used. Some studies have addressed the early detection of early molecular relapse or MRD using ctDNA, while a general interest existed in evaluating the potential of ctDNA-based MRD detection in predicting the treatment response and recurrence following adjuvant treatments, such as RT, CRT, or a combination with immunotherapy in HNSCC. A few studies using surgery with a curative intent have shown that ctDNA-based MRD detection is useful in predicting recurrence in patients with resectable HNSCC. ctDNA has also been shown to be clinically relevant in evaluating the response to RT in treatment-naive patients or to re-irradiation in patients with locally advanced HNSCC who have recurred. Several studies using combination therapies, such as surgery plus RT or CRT or CRT alone with plasma sampling during and after definitive treatment, demonstrate the prognostic value and absolute utility of ctDNA profiling in predicting recurrence in a substantial proportion of HNSCC patients with locally advanced disease. Treatments involving ICB-based immunotherapy have been used in patients with recurrent/metastatic HNSCC. In these studies, ctDNA was shown to be a reliable biomarker for assessing the efficacy of immunotherapy. The results of all these studies indicate that the ctDNA status during or after definitive treatment may be beneficial in modulating the intensity of adjuvant treatment; it could be deintensified in patients with undetectable ctDNA, and patients whose ctDNA testing suggests MRD after curative-intent therapy could potentially benefit from intensified adjuvant treatment.

Despite encouraging results on the clinical benefit of ctDNA in HNSCC, the studies included in this review have numerous limitations. The most significant limitation is the low number of patients enrolled in studies, which may have reduced the statistical power. Furthermore, most studies include a heterogeneous population, including different HNCC subtypes. Furthermore, most studies report unsatisfactory rates of sensitivity of ctDNA detection, partly due to the lack of sensitive techniques for mutation detection in plasma DNA. Many issues that need to be addressed in future studies include the standardization and harmonization of the analytical methods, platforms, and bioinformatic pipelines used; their proper validation; and the inclusion of reliable and meaningful quality measures, as well as clear descriptions of analytical sensitivity and specificity when applied in patient blood plasma—as outlined in national and international guidelines (e.g., RiliBäk 2024). In particular, it has to be described which and how many relevant genes are addressed, which variant allele frequency is detected, and whether tumor fraction, copy number variation, or fragmentomic scores are additionally assessed. A highly important issue is the evaluation of preanalytics and the recommendation of standard operating procedures for sample collection, processing, and storage.

From a clinical perspective, the optimal timing of serial venipunctures, choosing the most informative time points during and after therapy, and the definition of clinically relevant changes of individual ctDNA levels are essential for the interpretation of the findings. This has to be done for all relevant clinical questions, as the dynamics of tumor mass reduction and plasma ctDNA levels may differ for surgery, CRTs, and immunotherapies. Therefore, well-designed, prospective, multicenter validation studies are urgently needed to provide high-level evidence for the use of ctDNA in routine care. If these issues are addressed thoroughly, high-quality ctDNA diagnostics will become a valuable new tool that can be integrated into existing clinical decision algorithms for HNSCC—which include surgery, RT, systemic therapy, immunotherapy, or their combinations—and where there is an urgent need to determine MRD and predict early response and recurrence in a timely manner. Hopefully, this will contribute to improving therapy guidance and reducing mortality and morbidity in HNSCC patients in the future.

## Figures and Tables

**Table 1:** An overview of the studies evaluating ctDNA profiling in the treatment monitoring of HNSCC patients.

Tumor Sites	Method of ctDNA Analysis	Objective of Study	Treatment Modality	Main Findings	Reference
Oral cavity, oropharynx, larynx, hypopharynx (Total *n* = 17)	A sequencing-based personalized ctDNA assay (RaDaR^TM^) for mutation detection	Detection of MRD and recurrence	Surgery	Tumor-specific variants were detected in all pre-operative plasma samples. In patients who relapsed, ctDNA was detected before progression, making personalized ctDNA analysis feasible for detecting MRD and clinical recurrence	[34]
Oral cavity, oropharynx, larynx, hypopharynx (Total *n* = 43)	The targeted sequencing of a panel of genes	Relevance of ctDNA in MRD detection	Surgery	ctDNA-based MRD detection predicted clinical recurrence with a median lead-time of 9.9 months in 17 out of 27 patients. ctDNA in samples obtained within 14 weeks after surgery, correlated with disease recurrence	[35]
Oropharynx and non-HNC (lung and esophageal cancers) (Total *n* = 23)	The sequencing-based detection of copy number instability	MRD detection	Curative RT in most patients (total doses 30–68.4 Gy)	Copy number instability-based ctDNA score reflects tumor burden and is feasible for MRD detection	[36]
Oral cavity, oropharynx, nasal cavity, nasopharynx, hypopharynx, sinuses, and skull base (Total *n* = 16)	The whole-genome sequencing-based detection of copy number variation (CNV)	Correlation of ctDNA with disease outcome after re-irradiation in locally advanced HNSCC	Re-RT (51–60 Gy)	CNV-based ctDNA abundance reflects the initial response to re-radiation and recurrence	[37]
Oral cavity, larynx, oropharynx (Total *n* = 29)	Multiplex PCR NGS assay	Comparing PET and ctDNA for detection of high-risk patients after treatment	Surgery (34% of the patients) and definitive CRT (66%)	ctDNA detection after definitive treatment was associated with a higher risk of disease recurrence, with 100% specificity at a sensitivity rate of 78%	[38]
HNSCC (oropharynx, tonsillar carcinoma, base of tongue and hypopharynx) and oligometastatic disease with various primary tumors (Total *n* = 28)	The low-pass, whole-genome sequencing-based detection of copy number alterations and fragment length distribution	Monitoring of treatment response in locally advanced HNSCC and oligometastatic disease	High-dose RT (70 Gy) and cisplatin	ctDNA analysis based on copy number alterations is useful for the detection of progression following RT	[39]
HPV-negative locoregionally confined HNSCC (Total *n* = 30)	The personalized sequencing-based mutation detection and sequencing of immunoprecipitated methylated plasma DNA	Predicting disease recurrence in HPV-negative locoregionally confined nonmetastatic HNSCC	Surgery in all patients, plus RT or CRT in most patients	Higher ctDNA abundance after treatment compared to baseline is indicative of disease recurrence	[40]
Oral cavity, larynx (Total *n* = 28)	Personalized commercial ctDNA test	Detection of minimal residual disease	All patients underwent surgical resection. The majority of high-risk patients received adjuvant CRT	The rate of disease recurrence was higher in patients with high-risk features and correlated with ctDNA	[41]
Mostly oropharynx (Total *n* = 20)	Exosome sequencing of 71 genes frequently mutated in HNC	Predicting recurrence	Surgical resection in all patients; CRT in 11 patients	During follow-up, no ctDNA was detected in 13 recurrence-free cases, but it was detected in 5 of the 7 recurrent cases; relapse-free survival time is significantly shorter in those with ctDNA detection post-treatment	[42]
Oropharynx, hypopharynx, oral cavity (Total *n* = 20)	The FFPE-tumor tissue informed sequencing of 127 driver genes in plasma	Prediction of disease recurrence	Definitive CRT with cumulative radiation dose of 70–77 Gy and concomitant CT with cisplatin weekly or a combination therapy of 5-fluorouracil and mitomycin C	In patients with ctDNA detection at the first follow-up point, disease recurrence occurred. All of the patients with detectable MRD suffered a relapse	[43]
Oropharynx, larynx, oral cavity, hypopharynx (Total *n* = 32)	Tumor-informed RaDaR^®^ assay and a tumor-naive CAPP-seq assay	Impact of ctDNA analysis in MRD detection in locally advanced HNSCC patients	Curative intent (i) surgery followed by adjuvant CRT (ii) definitive RT or (iii) CRT, with CRT being the most frequent (78%) treatment	ctDNA detection during follow-up was associated with shorter relapse-free survival (*p* < 0.001). RaDaR assay proved to be better in MRD detection than CAPP-seq assay	[44]
Oral cavity, oropharynx, hypopharynx, larynx, the external auditory canal (Total *n* = 26)	The tumor-informed monitoring of an HNSSC-related panel of genes (*n* = 31) in plasma by digital PCR	Monitoring treatment response and relapse in HNSCC	Of the patients, 1 was treated with RT, 9 with surgery, 5 with CRT, 3 with induction CT and CRT, 2 with induction CT and surgery, 2 with surgery plus CRT and 3 with induction CT, surgery, and CRT	In cases with relapse, the ctDNA reverted to positive or did not become negative after the initial curative treatment. Patients who remained ctDNA negative after initial curative treatment were alive without recurrence and had a significantly better prognosis than those who reverted to ctDNA positivity	[45]
Oral cavity, oropharynx, larynx, hypopharynx, and others (paranasal sinus, nasopharyngeal, and salivary gland cancers, unknown primary) (Total *n* = 116)	The sequencing of a panel of 26 genes, including the most-frequently mutated genes in HNSCC and two HPV-16 genes	Utility of ctDNA to detect MRD in unselected locally advanced HNSSC	Most patients (92.4%) received curative-intent CRT as the primary treatment while the remaining patients (7.6%) were treated with surgery	Median 2-year PFS rate was 23.53% and 86.6% in MRD-positive and MRD-negative patients. Median survival was 28.37 months for MRD-positive patients and was not reached for the MRD-negative cohort (*p* = 0.011)	[46]
Larynx, oropharynx oral cavity, hypopharynx nasopharynx parotid gland, neck (Total *n* = 38)	The sequencing of a panel of approximately 70 genes at baseline and after the completion of treatment	Feasibility of ctDNA in identifying high-risk patients	CRT	In the patients with complete or partial response to CRT, less mutations (26.6%) were observed than in nonresponders (75%) (*p* = 0.03). Assessment of mutations before and after CRT is helpful to characterize patients with a high risk of locoregional recurrence or metastatic progression	[47]
Oral cavity, oropharynx, hypopharynx, larynx, HNC unknown primary (Total *n* = 97)	A tumor-agnostic sequencing assay including 37 genes frequently mutated in recurrent/metastatic HNSCC	Predictive value of ctDNA profiling in the efficacy of single-agent PD1 inhibitor in recurrent/metastatic HNSCC	Treatment with non-curative intent anti-PD1 therapy (pembrolizumab or nivolumab) without concomitant CTx	Among the 35 patients with ctDNA positivity at baseline, 17 had a decrease (negative ΔctDNA) in the mean VAF and the majority of them (76.4%) achieved either a complete response, partial response, or stable disease, with 4 cases experiencing disease progression. Among those with an increase (positive ΔctDNA) in the mean VAF, the majority of the cases (72%) had progressive disease	[48]
Oral cavity (the tongue, buccal mucosa, gingiva, and other oral locations) (Total *n* = 12)	The targeted sequencing of 74 genes	ctDNA as real-time biomarker for monitoring response to ICB therapy in HNSCC	Nivolumab was second-line therapy for 3 patients who were refractory to platinum-based CTx, whereas the other 7 patients received pembrolizumab as first-line therapy	Changes in the mean VAF, detected in the early phase of the treatment course, often preceded radiological progression. Among patients with an achieved partial response or stable disease, mean VAF remained relatively stable whereas in the patients with progressive disease, early increases in the mean VAF occurred and accurately predicted disease progression	[49]
Oral cavity, oropharynx, larynx, hypopharynx, nasal cavity (Total *n* = 53)	Sequencing a panel of genes (CAPP-seq) optimized for HNSCC	Monitoring of immune checkpoint blockade therapy in metastatic HNSCC	Patients received one or more cycles of first-line, platinum-based, systemic treatment (*n* = 16); second-line anti-PD1/PL1 monotherapy (*n* = 30); or combination immunotherapy with two agents (*n* = 7)	Baseline ctDNA was not informative for OS or PFS. However, a change in ctDNA VAF after one cycle of treatment, compared to baseline (ΔVAF), was predictive of both PFS (*p* < 0.01) and OS (*p* < 0.01). A decrease in ΔVAF identified patients with longer OS despite early radiological progression.	[50]

MRD, minimal residual disease; RT, radiotherapy; CTx, chemotherapy; CRT, chemoradiotherapy; ICB, immune checkpoint blockade.
